# DFT Exploration of Metal Ion–Ligand Binding: Toward Rational Design of Chelating Agent in Semiconductor Manufacturing

**DOI:** 10.3390/molecules29020308

**Published:** 2024-01-08

**Authors:** Wenyuan Wang, Junli Zhu, Qi Huang, Lei Zhu, Ding Wang, Weimin Li, Wenjie Yu

**Affiliations:** 1 School of Materials and Chemistry, University of Shanghai for Science and Technology, Shanghai 200093, China; 213353185@st.usst.edu.cn (W.W.);; 2 State Key Laboratory of Functional Materials for Informatics, Shanghai Institute of Microsystem and Information Technology, Chinese Academy of Sciences, Shanghai 200050, China; huangqi@mail.sim.ac.cn (Q.H.); weimin.li@mail.sim.ac.cn (W.L.); 3 Shanghai Institute of IC Materials Co., Ltd., Shanghai 201899, China; junli.zhu@sicm.com.cn

**Keywords:** microelectronic process, chelating agent, quantum chemical calculations, metal ion, ligand, deprotonation

## Abstract

Chelating agents are commonly employed in microelectronic processes to prevent metal ion contamination. The ligand fragments of a chelating agent largely determine its binding strength to metal ions. Identification of ligands with suitable characteristics will facilitate the design of chelating agents to enhance the capture and removal of metal ions from the substrate in microelectronic processes. This study employed quantum chemical calculations to simulate the binding process between eleven ligands and the hydrated forms of Ni^2+^, Cu^2+^, Al^3+^, and Fe^3+^ ions. The binding strength between the metal ions and ligands was quantified using binding energy and binding enthalpy. Additionally, we explored the binding interaction mechanisms and explained the differences in binding abilities of the eleven ligands using frontier molecular orbitals, nucleophilic indexes, electrostatic potentials, and energy decomposition calculations based on molecular force fields. Based on our computational results, promising chelating agent structures are proposed, aiming to guide the design of new chelating agents to address metal ion contamination issues in integrated circuit processes.

## 1. Introduction

Metal impurities in silicon substrates are a critical factor that must be rigorously controlled during silicon wafer fabrication. Metal particles in ionic form exhibit high mobility within semiconductor silicon substrates, which can cause significant damage to the electrical performance and long-term reliability of silicon-based semiconductor devices [1,2,3].

Stringent control measures are essential at various stages of silicon wafer fabrication processes. Currently, the most common and effective approach is to incorporate chelating agents into the liquid environment during the fabrication process. Chelating agents enable metal ions to exist in the form of chelate complexes, effectively preventing metal ion contamination.

Chelating agents are composed of multiple ligands with electron-donating properties. These ligands coordinate with metal ions through their electron-donating atoms, resulting in the formation of highly stable cyclic chelates that resist dissociation [4]. In the realm of integrated circuits and microelectronics, chelating agents play a critical role and find widespread applications in various processes, including substrate polishing, cleaning, and even thin film layer etching [5,6].

As integrated circuit fabrication processes continue to advance, the requirements for reducing metal contamination on silicon substrate surfaces are raised. The demand for lower surface metal concentrations decreases by approximately one order of magnitude every decade [7]. By the 2010s, to manufacture 1 Gb DRAM, the criteria necessitated silicon wafer surfaces to exhibit metal impurity concentrations lower than 1.0 × 10^9^ atom/cm^2^ [7]. Presently, the pursuit of enhanced metal impurity cleanliness has emerged as a paramount challenge within the realm of integrated circuit fabrication processes.

Hence, within various processes of semiconductor silicon wafer fabrication, researchers and industry professionals are diligently engaged in the detection, control, reduction, elimination, and prevention of metal impurities. This places higher demands on chelating agents used in semiconductor manufacturing. Presently, the selection of chelating agents in semiconductor processes is predominantly guided by experimental screening. For instance, Fujimi Corporation [8,9] applied amino carboxylic acids and amino-phosphorous acid acid-based chelating agents in chemical mechanical polishing processes for silicon wafers. Hyun Soo Roh [10] proposed a series of carboxylic acid chelating agents for precision polishing. Additionally, Liu Yuling introduced specialized chelating agent FA/O for microelectronics and conducted research on its applications and impacts in silicon wafer polishing [5], cleaning [11], copper interconnect layer polishing [12], as well as improving the electrochemical corrosion between cobalt and copper in copper interconnect layer polishing processes [13]. The aforementioned chelating agents share analogous functional ligands, such as carboxyl groups, phosphorous acid groups, hydroxyl groups, amino groups, and others. However, there is a paucity of theoretical investigations and empirical evidence to comprehensively elucidate their mechanisms of action and the underlying rules governing their chelation strengths.

Quantum chemical calculations extensively investigate the characteristics and mechanisms of interactions between metal ions and ligands. Leonardo M. da Costa et al. [14] conducted a comprehensive analysis involving geometric, electronic, and energetic parameters to quantitatively assess the coordination affinities of 32 ligands, including phosphine, amine, and thiocarbonyl ligands, with [Ni(H_2_O)_5_]^2+^ complex. The ligand coordination strengths were found to follow the order of carbonyl < thiocarbonyl < amine < phosphine. Daniel S. G. Quattrociocchi et al. [15] computed the affinities of pentahydrated-Ni^2+^ ions for 16 different neutral ligands with distinct functional groups. Ligands containing the P=O structure exhibited the highest binding affinity, and their coordination bonds with nickel ions had a significant electrostatic component. This conclusion is valuable for optimizing chelating agents used in the treatment of wastewater containing nickel ions. Marcos. V. M. Meuser et al. [16] investigated the affinities of three-hydrated Pb^2+^ ions for 14 different ligands. The results indicate that the interaction between phosphorus oxide ligands and metal cations is the strongest. Next are ligands with double bond structures, and finally, ligands containing single bond structures. Sambath Baskaran et al. [17] conducted an analysis of the bonding properties and chemical hardness of Cu(III) alkyl complexes. They also performed energy decomposition analysis (EDA) calculations on the complexes to explore the relationship between the stability of the complexes and their bonding components. The conclusion drawn is that the stabilization energy of the Cu (III)–Et*_trans_* bond is relatively higher, the bond order is also higher, and it is more ionic in nature.

R. López et al. [18] presented a comprehensive computational database of the complexes involving alkali metal cations (Li^+^, Na^+^, K^+^) and alkaline earth metal cations (Be^2+^, Mg^2+^, and Ca^2+^). This database includes accurate geometric structures and binding energies for interactions between these metal cations and the 25 small ligands with different charges and donor atoms (“O”, “N”, and “S”). The results were rigorously validated against experimental data, providing valuable insights into the interactions of metal cations with ligands in proteins and nucleic acids. Similar studies on the interaction strength between ligands and metal ions have also been reported in other works [19,20].

However, these studies have certain limitations in the application of microelectronics processes. Firstly, they did not account for the deprotonation behavior of the ligands. In the application scenarios of chelating agents in microelectronics processes, the environment is often alkaline, leading to deprotonation of the ligands’ acidic side groups. Research in this aspect is currently lacking. Secondly, metal ion contamination in microelectronics processes is not limited to a single metal ion. It typically involves various metal ions such as iron, nickel, copper, and aluminum. The differences in binding mechanisms between different types of metal ions and ligands remain unclear and warrant a comparative investigation. Therefore, this study explores the binding characteristics and underlying driving forces of 11 ligands, including four deprotonated acidic ligands featuring various functional groups. Among these ligands, some are recognized as “chelators” that have been applied in microelectronic processes, but their mechanisms are not clear. Other ligands are selected based on previous studies on chelating agents in different fields or our own rational choices. The research delves into the binding characteristics and potential driving forces in the interactions of these ligands with Ni^2+^, Cu^2+^, Al^3+^, and Fe^3+^—representative metal cations notorious for causing failures in silicon-based semiconductor devices. Quantum chemical calculations were conducted to quantify binding strength through binding enthalpy and binding energy calculations. In addition, we analyzed the frontier molecular orbitals, nucleophilic indexes, and electrostatic potentials of the ligands, as well as the electrostatic potential of the resulting coordination complexes. We also introduced energy decomposition calculations based on molecular force fields to examine the nature of the interactions between ligands and metal ions, thereby revealing the fundamental driving forces in the binding process. Finally, promising chelating agent structures are proposed, which exhibit the strongest binding affinity with each of the four metal cations.

## 2. Calculation Methods

All calculations were performed using the density functional theory (DFT) within the Gaussian16 [21] and GaussianView6 [22] software. The TPSSH [23,24] functional combined with the DFT-D3 [25] correction was employed. For structure optimization, the SDD basis set [26,27] was used for the metal ions, while the def2tzvp [28] basis set was used for other atoms (C, N, O, H, S, P). Single-point energy calculations were carried out using the def2tzvpp [28] basis set. These methods have been demonstrated to effectively describe the interactions between Ni/Cu/Al/Fe ions and organic molecules [29,30,31,32].To simulate the molecular systems in a solvent-like environment, the calculations were performed using the polarizable continuum model (PCM) [33] for solvation. Additionally, vibrational frequency calculations were performed to ensure the absence of imaginary frequencies and confirm that the optimized structures correspond to the true energy minima. For the analysis of the system’s frontier molecular orbitals [34] and electrostatic potential [35], we performed calculations and processing using Multiwfn [36]. The obtained data were then visualized using VMD [37].

**Figure 1:**
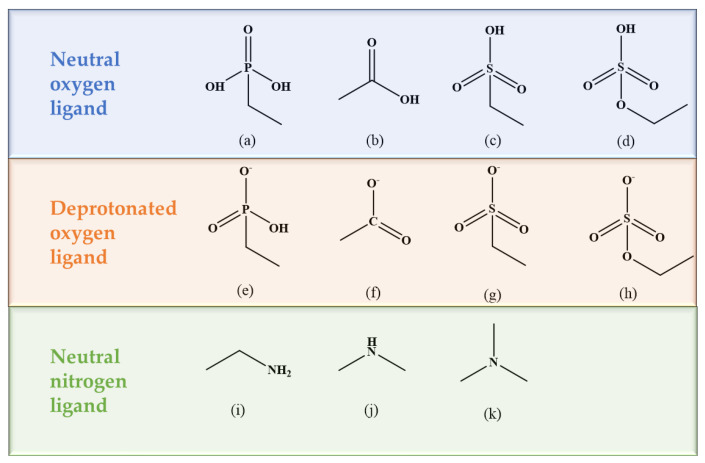
Ligand diagram:(**a**) phosphorous acid (RPO_3_H_3_), (**b**) carboxylic acid (RCOOH), (**c**) sulfonic acid (HSO_3_R), (**d**) sulfuric acid (HSO_4_R), (**e**) phosphorous acid ion (RPO_3_^−^H_2_), (**f**) carboxylic acid ion (RCOO^−^), (**g**) sulfonic acid ion (HSO_3_^−^R), (**h**) sulfuric acid ion (HSO_4_^−^R), (**i**) amine (RNH_2_), (**j**) dimethylamine (R_2_NH), (**k**) trimethylamine (R_3_N).

Three types of ligands were investigated in this study, as depicted in Figure 1. Four acidic ligands, including phosphorous acid, carboxylic acid, sulfonic acid, and sulfuric acid, as well as their deprotonated forms (the deprotonation process is mentioned in References [38,39]), are shown in Figure 1a–h. Additionally, three amine ligands containing C-N single bond shown in Figure 1i–k, namely, amine, dimethylamine, and trimethylamine, were also examined.

**Figure 2:**
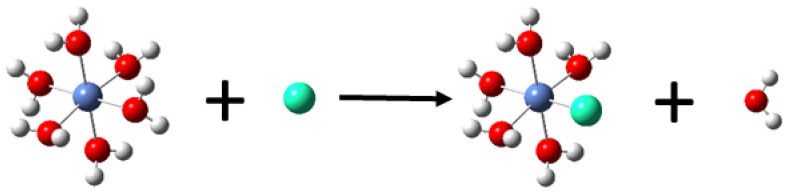
Substitution reaction for the change of a water molecule in the nickel ion hydrate by the ligands shown in Figure 1.

To better mimic the aquatic environment, this study employed fully hydrated metal ions for binding simulations, as shown in Figure S1. Specifically, these included a hexa-coordinated octahedral nickel ion hydrate, a tetra-coordinated square planar copper ion hydrate, a hexa-coordinated octahedral trivalent aluminum ion hydrate, and a hexa-coordinated trivalent iron ion hydrate. Following testing, each metal ion adopted its respective most stable spin state (Ni^2+^ as a triplet, Cu^2+^ as a doublet, Al^3+^ as a singlet, and Fe^3+^ as a sextet). The binding mode of the nickel ion hydrate is depicted as Equation (1) (where *U* denotes the ligand) and illustrated in Figure 2. In this mode, a monodentate ligand molecule replaces a water molecule from the nickel ion hydrate, and the electron-deficient atom in the ligand occupies the position originally held by the oxygen atom of the water molecule, forming a coordination bond with the metal ion. The other three metal ions follow a similar binding mode, as described in Equations (2)–(4):

(1) $${\left[Ni{\left(H_{2}O\right)}_{6}\right]}^{\left(2+\right)}+U\to {\left[Ni{\left(H_{2}O\right)}_{5}U\right]}^{\left(2+\right)}+H_{2}O$$

(2) $${\left[Cu{\left(H_{2}O\right)}_{4}\right]}^{\left(2+\right)}+U\to {\left[Cu{\left(H_{2}O\right)}_{3}U\right]}^{\left(2+\right)}+H_{2}O$$

(3) $${\left[Al{\left(H_{2}O\right)}_{6}\right]}^{\left(3+\right)}+U\to {\left[Al{\left(H_{2}O\right)}_{5}U\right]}^{\left(3+\right)}+H_{2}O$$

(4) $${\left[Fe{\left(H_{2}O\right)}_{6}\right]}^{\left(3+\right)}+U\to {\left[Fe{\left(H_{2}O\right)}_{5}U\right]}^{\left(3+\right)}+H_{2}O$$

**Figure 3:**
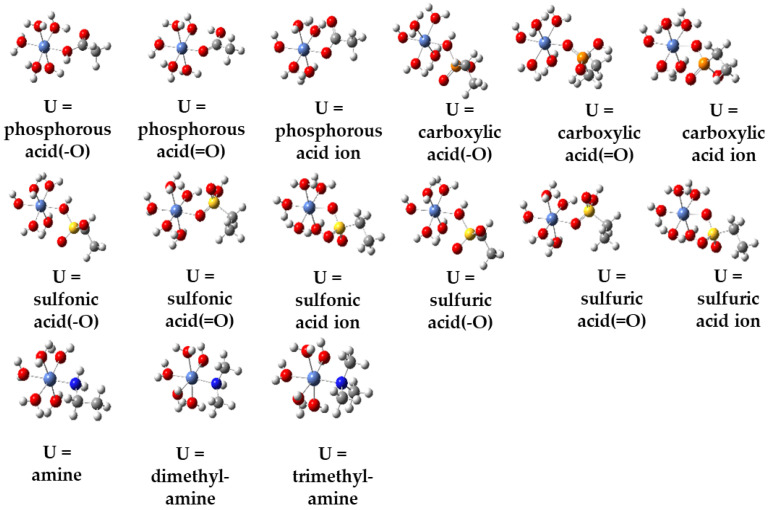
Computational geometries of the binding products of nickel ion bound to 11 ligands, totaling 21 binding systems (U denotes ligand).

The optimized structures of metal ion hydrates are depicted in Figure S1. The coordination bond lengths of the post-optimization structures of the four metal ion hydrates closely resemble the theoretical values, indicating the accuracy of our computational methods (refer to Table S1). The optimized structure of the coordination compound of the nickel ion with the ligands is shown in Figure 3, and the optimized structures of the remaining coordination compounds are shown in Figure S2. Following structural optimization, there is a slight displacement in the ligand positions. However, the relative positions between the coordinating atoms and the metal atoms remain essentially unchanged. In other words, the geometric shape of the atoms surrounding the metal cation remains mostly unaltered.

### 2.1. Binding Strength

We used binding enthalpy and binding energy as metrics to measure the strength of chemical interactions in our study.

Binding enthalpy (∆*H_binding_*) has been used to quantify the affinity between metal ions and ligands [14,19,39,40]. Binding enthalpy reflects the magnitude of non-covalent interactions such as hydrogen bonds and van der Waals forces between molecules. It can be used to evaluate the affinity and selectivity between ligands, metal ions, and solvents [41]. In our study, we have utilized thermally corrected computations to determine binding enthalpy at 298 K, which accurately represents the affinity between metal ions and ligands. The formula for calculating binding enthalpy is as follows (Equation (5)):

(5) $$\Delta H_{binding}=H_{compound}+H_{ion\_hydration}-(H_{water}+H_{ligand})$$

∆*H_binding_* represents the enthalpy change associated with the binding process, where *H_compound_*, *H_ion_hydration_*, *H_water_*, and *H_ligand_* denote the enthalpies of the complex formation, ion hydration, water molecule, and ligand, respectively. A negative ∆*H_binding_* indicates a preferred affinity of the metal cation for the monodentate ligand compared to water molecules.

Binding energy refers to the energy released when two or more chemical particles (atoms) undergo a binding reaction to form new particles. The magnitude of binding energy is a key metric specific to binding (chemical) reactions, used to quantify the ease or difficulty of the occurrence of a reaction. Since the energy changes in the systems described in this paper result from electronic interactions, electronic energy is employed as a measure of binding energy. The formula for calculating binding energy in the context is provided in Equation (6):

(6) $${\Delta E}_{binding}=E_{compound}+E_{ion\_hydration}-(E_{water}+E_{ligand})$$

*E_compound_*, *Ei_on_hydration_*, *E_water_*, and *E_ligand_* represent the system energies of the substituted complex, hydrated metal ion, water molecule, and ligand, respectively. ∆*E_binding_* denotes the energy change during the combination process, which reflects the strength of the binding between the ligand and the metal ion. 

### 2.2. Frontier Molecular Orbitals (FMOs) and Energy Gaps

Frontier molecular orbitals refer to the highest occupied molecular orbital (HOMO) and the lowest unoccupied molecular orbital (LUMO) within a molecule. The HOMO typically exhibits a relatively loose electron-binding nature, characterized as an electron-donating entity. Conversely, the LUMO possesses a strong electron affinity, serving as an electron-accepting entity. These two orbitals play an extremely crucial role in chemical reactions [42].

The ∆*E* (*E_LOMO_-E_HOMO_*) energy gap represents the energy difference between the HOMO and LUMO orbitals, which is a critical reference value for the reactivity of chemical reactions.

### 2.3. The Electrophilicity ω Index and the Nucleophilicity N Index

The electrophilicity index and nucleophilicity index are among the commonly used analysis methods in Conceptual Density Functional Theory (DFT) [43,44]. The electrophilicity index (*ω*) is related to the molecule’s strong electron-accepting ability, while the nucleophilicity index (*N*) is the opposite, related to the molecule’s strong electron-donating ability [45]. The electrophilicity *ω* scale allowed the classification of organic molecules as strong electrophiles with *ω* > 1.5 eV, moderate electrophiles with 0.8 < *ω* < 1.5 eV, and marginal electrophiles with *ω* < 0.8 eV [46], akin, the nucleophilicity *N* scale allowed a further classification of organic molecules as strong nucleophiles with *N* > 3.0 eV, moderate nucleophiles with 2.0 < *N* < 3.0 eV, and marginal nucleophiles with *N* < 2.0 eV [47].

The local electrophilicity *ω_k_* [48] and the local nucleophilicity *N_k_* [49] indexes, which permit the distribution of the global electrophilicity ω and nucleophilicity N indexes at the atomic sites k, enable the activity information of individual atoms in the molecule to be examined very clearly. Numerous experimental and theoretical studies have proven the feasibility of these local descriptors to study regio- and chemoselectivities [43,44]. This paper examines the nucleophilic nature of ligand molecules. 

### 2.4. Electrostatic Potential

The electrostatic potential refers to the work done when moving a unit of positive charge from infinity to a specific point in the space surrounding a molecule. It can also be seen as the interaction energy between a unit positive charge located at a point r and the current system [50]. Its calculation formula is given by Equation (7) [51]:

(7) $$v_{tot}(r)=v_{nuc}(r)+v_{ele}(r)=\sum_{A}\frac{Z_{A}}{\left|r-R_{A}\right|}-\int \frac{\rho (r^{\prime })}{\left|r-r^{\prime }\right|}dr^{\prime }$$

The electrostatic potential is composed of two parts: the nuclear charge of atom A (denoted as *Z*) and the electron density (denoted as *ρ*). The former contributes a positive value, while the latter contributes a negative value. A positive (or negative) electrostatic potential indicates that the potential is dominated by the charge of the nucleus (electrons) at that point. The electrostatic potential distribution on molecular van der Waals surfaces has long been used to analyze the charge distribution of various organic molecules [52,53], organic molecules interacting with metals, and inorganic molecular systems [53]. It is employed to predict properties such as reaction sites, aiding researchers in gaining a deeper understanding of the nature of chemical reactions and interactions between molecules.

### 2.5. Energy Decomposition Analysis Based on Forcefield (EDA-FF) 

Energy decomposition is a crucial component of quantum chemical calculations. It allows for the total inter-fragment interaction energy to be decomposed into physically meaningful energy terms, facilitating the examination of the nature of interactions. In fact, compared to mainstream wave function-based energy decomposition methods such as Morokuma and SAPT, molecular force fields (forcefield), which are based on a very simple form, can also decompose components of weak interactions. This is referred to as energy Decomposition Analysis based on forcefield (EDA-FF) [54]. 

In molecular force fields, non-bonding interactions include electrostatic interactions and van der Waals forces. The latter can be divided into a “repulsion term” that causes repulsion and a “dispersion term” that causes attraction. This method is extremely time-efficient and yields results with clear physical meanings. In many cases, it can replace more expensive wave function-based energy decomposition methods and can conveniently examine weak interactions within molecules qualitatively. Currently, many articles have used the EDA-FF method to study problems and have obtained meaningful results [55,56,57,58,59,60]. In the present study, the more precise AMBER force field [61] was used to probe the composition of the metal center and ligand interactions.

## 3. Results

We quantified the binding strength between metal ions and ligands using binding energy and enthalpy. The binding enthalpies and energies for the substitution processes involve four types of metal ion hydrates and ligands, which are summarized in Tables S2 and S3. 

**Figure 4:**
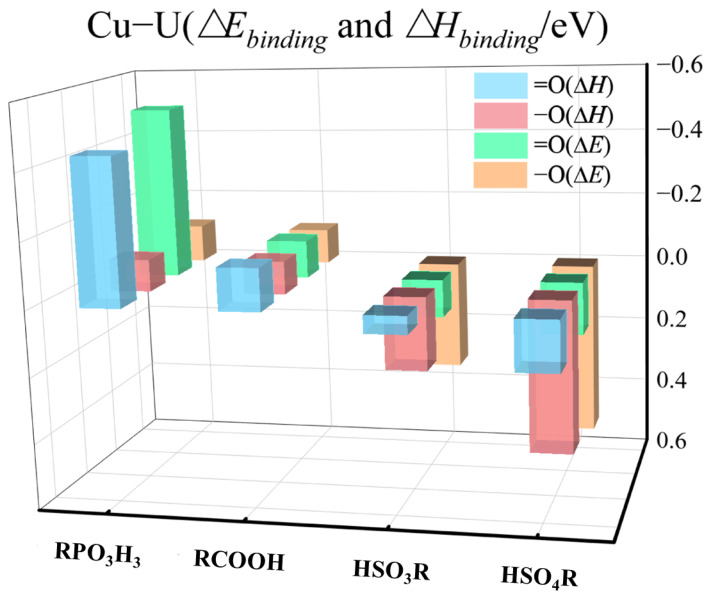
Bar chart of the binding enthalpies and binding energies between copper ions and four ligands at single and double-bond oxygen sites.

N and O atoms are often the electron-donating atoms in the ligand. Some of the ligands in this study system contain both single-bonded oxygen and double-bonded oxygen structures. In order to clarify the functions of the single-bonded oxygen and double-bonded oxygen in the binding process, we separately performed binding simulations for them. Figure 4 presents the bar graph of the binding enthalpies and binding energies, respectively, for copper ions with the four ligands at their single and double-bond oxygen sites. The results indicate that, prior to deprotonation, the double-bond oxygen sites of the ligands exhibit stronger binding with copper ions than the single-bond oxygen sites. Similar results can be found in other studies [12,40,51]. The binding characteristics of the single-bonded and double-bonded oxygen atoms with the other three metal ions also show similar trends, as shown in Figure S3.

**Figure 5:**
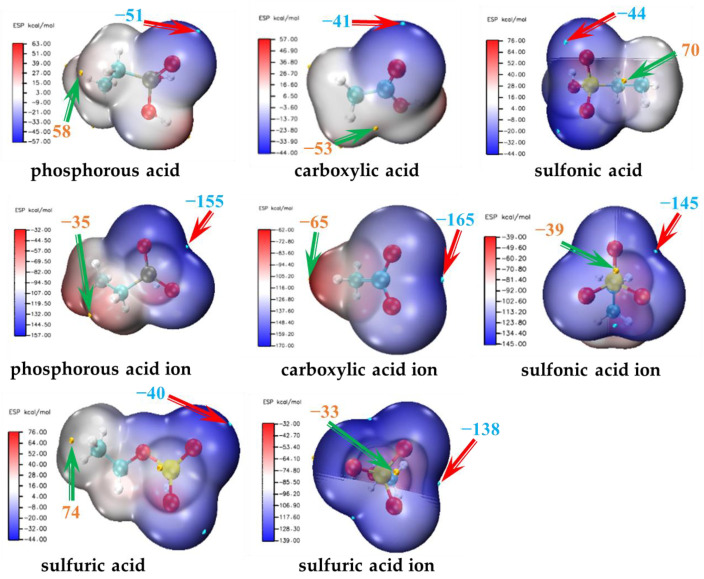
Distribution of electrostatic potential for oxygen-containing ligands before and after deprotonation (where the red arrow points to the point of ligand electrostatic potential minimum and the green arrow points to the point of electrostatic potential maximum).

We visualized the electrostatic potential of the oxygen-containing ligands before and after deprotonation in Figure 5 (the remaining nitrogen ligand electrostatic potential visualization is shown in Figure S4). As can be seen from the figure, before deprotonation, the minimum point of the ligand’s electrostatic potential is located on the van der Waals surface of the double-bond oxygen, indicating a stronger electron loss potential at the double-bond oxygen site. After deprotonation, however, the minimum point of the ligand’s electrostatic potential is located at the intersection of the van der Waals surfaces between single and double-bond oxygen, suggesting similar electron loss tendencies at both sites. Figure S5 displays the local electrophilicity and nucleophilicity indexes, reduced to each atom, for eleven ligands. Similar to the results of the electrostatic potential, in neutral oxygen ligands, the atom with the highest nucleophilicity index is always the double-bonded oxygen atom of the ligand. After deprotonation, the nucleophilicity index of the deprotonated oxygen atom in the oxygen ligand increases sharply, and the difference in the nucleophilicity indexes of its single and double-bonded oxygen is very small. Therefore, we used the double-bond oxygen site for binding simulations of the neutral ligands and switched to using the deprotonated single-bond oxygen site for binding simulations of deprotonated ligands. This approach will be consistently applied in subsequent results and discussions without further elaboration.

**Figure 6:**
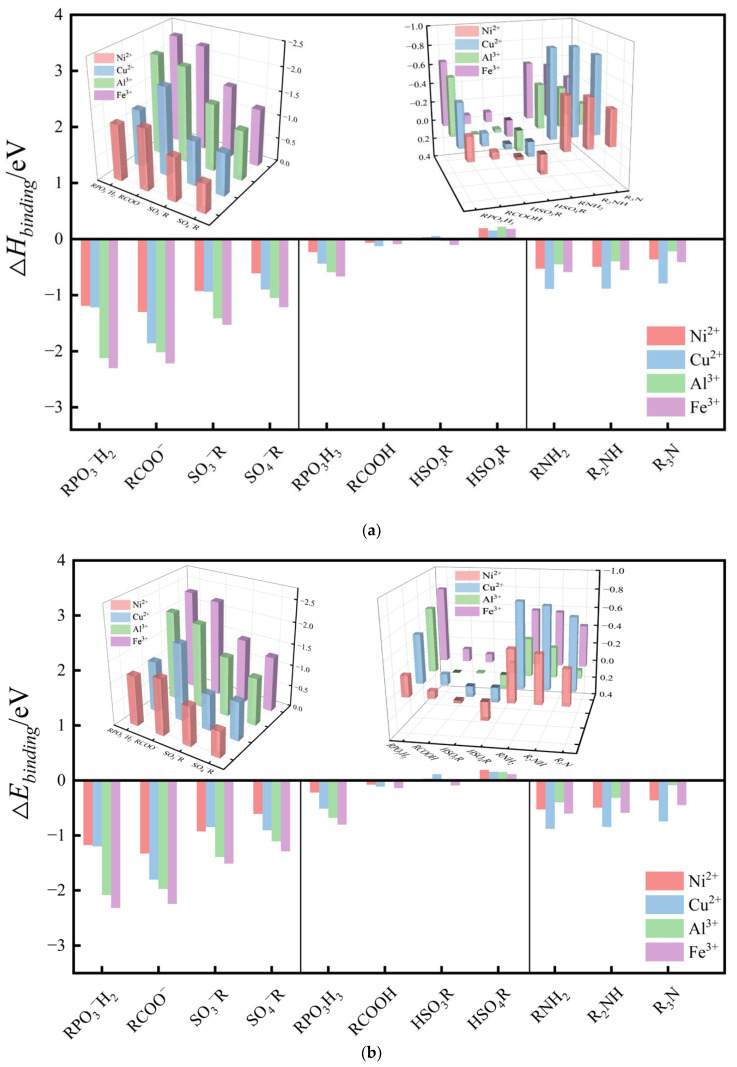
(**a**) Bar chart of binding enthalpies of four metal ions for the occurrence of the binding process with the ligand in Figure 1, (**b**) Bar chart of binding energies of four metal ions for the occurrence of binding processes with the ligands in Figure 1.

In microelectronic processes, chelating agents can be used in various chemical products, such as acidic or alkaline slurries and cleaning agents. Therefore, both the effects of ligand types and deprotonation have been explored in this study. Figure 6a,b show the binding enthalpies and binding energies, respectively, of the eleven ligands in this study for the hydrides of four metal ion hydrides. As shown in the figure, anionic ligands, including deprotonated phosphoric acid, carboxylic acid, sulfonic acid, and sulfuric acid, show very high binding strength with metal ions, significantly superior to other non-deprotonated ligands (increase in the binding energy by more than 1 eV). For the divalent ion Cu^2+^, the binding strength of the four anionic ligand acids is in the order carboxylic acid ion > phosphoric acid ion > sulfuric acid ion > sulfonic acid ion. For the trivalent metal ion Fe^3+^, the binding strength of phosphite exceeds that of carboxylate, with the order being phosphoric acid ion > carboxylic acid ion > sulfonic acid ion > sulfuric acid ion. For all four types of metal ions, phosphoric acid ion and carboxylic acid ion ligands have much higher binding strengths than the sulfonic acid ion and sulfuric acid ion.

Among the neutral ligands, significant differences can be found in their binding features. For oxygen ligands, as shown in Figure 6a,b, phosphite ligands exhibit strong binding with metal ions. The binding strength of the remaining three oxygen ligands with the studied metal ions is not significantly better than that of a water molecule. In fact, sulfate ligands are noticeably inferior to water molecules. Some oxygen ligands binding systems have inconsistent trends in binding enthalpy and binding energy (A complex system involving neutral carboxylate ligands, sulfate ligands, and deprotonated sulfonate ligands), which may be due to relatively high reaction barriers for these systems.

All three nitrogen-containing ligand systems exhibit high binding strength. Compared to oxygen ligands, nitrogen ligands have exceptionally high binding strength with copper ions. In fact, the binding strength of Cu^2+^ with amine even surpasses its binding strength with deprotonated sulfonic acid ligand, which may reflect its selectivity to some extent. The binding strengths of the three nitrogen ligands with metal ions are ranked as follows: amine > dimethylamine > trimethylamine. The binding strength decreases as the number of hydrogen atoms connected to the nitrogen atom decreases. This trend may be due to the -H structure enhancing the charge on the electron-losing N atom, which is consistent with the previous research results [40,51].

There are some differences in the binding trends between trivalent and divalent metal ions with ligands. For oxygen ligands, the binding strength with trivalent metal ions is generally higher than that with divalent metal ions. This is particularly true for the four deprotonated ligands: phosphorous acid ion, carboxylic acid ion, sulfonic acid ion, and sulfuric acid ion. Their binding energies with Fe^3+^ are 1.12, 0.444, 0.664, and 0.382 eV higher than those with Cu^2+^, respectively. 

## 4. Discussion

In order to explore the potential mechanisms influencing binding strength, we have linked the binding strength of metal ions and ligands with frontier molecular orbitals, nucleophilic indexes, and electrostatic potentials and conducted energy decomposition analysis based on forcefield on some binding systems.

### 4.1. Effect of Deprotonation on Binding Properties of Ligand

**Figure 7:**
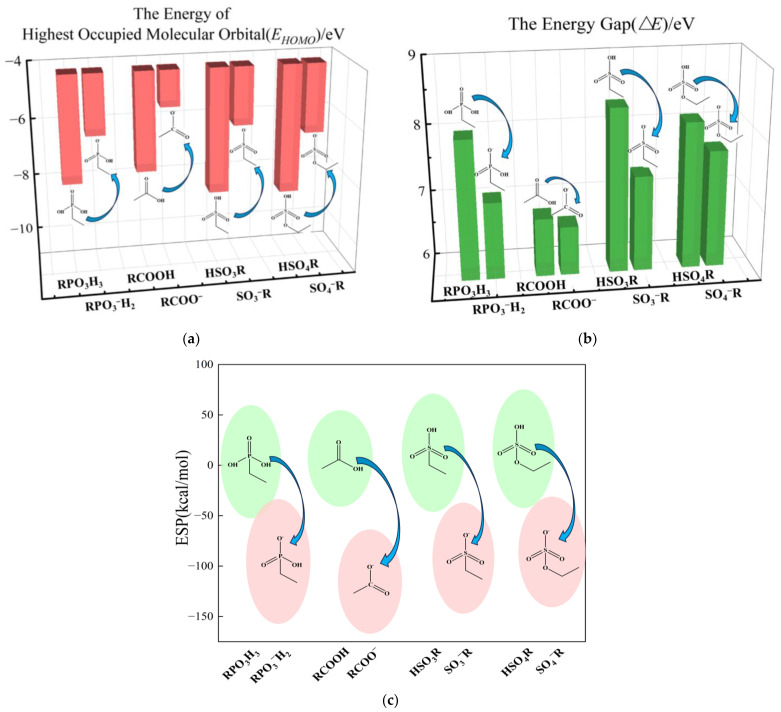
(**a**) Graph of the change in the energy of the highest occupied molecular orbitals (HOMO) of ligands before and after deprotonation, (**b**) Histogram of the energy gap of ligands before and after deprotonation, (**c**) Graph of the change in electrostatic potential of ligands before and after deprotonation.

The computational results from the previous section revealed that the deprotonation process has a significant impact on the binding ability of the ligands. Consequently, we employed frontier molecular orbitals and molecular electrostatic potential for an in-depth exploration of its mechanism. We have calculated and summarized the frontier molecular orbitals energy values and energy gaps (∆*E*) of the ligands as shown in Table S4. Figure 7a,b depict the trend graphs of the energy values of the highest occupied molecular orbitals (HOMO) and the energy gaps (∆*E*) of the ligands before and after deprotonation, respectively. High HOMO orbital energy values are associated with high electron-donating properties, while small energy gap values are related to stronger reactivity. The results show that after deprotonation, the *E_HOMO_* values of the four ligands increase by 2.15, 1.59, 2.30, and 2.05 eV, respectively, while the energy gaps (∆*E*) decrease by 0.14, 0.92, 1.00, and 0.43 eV, respectively. These significant differences indicate that after deprotonation, the ability of the ligands to bind electrons in their HOMO orbitals weakens, and their reactivity increases, making them more prone to electron loss and nucleophilic substitution reactions.

We have calculated the electrostatic potential of the ligands, and the results are shown in Table S5. Figure 7c presents a schematic representation of the changes in the electrostatic potential of the ligands before and after deprotonation. The results show that after deprotonation, the electrostatic potential of the ligands significantly decreases, with all values falling into the negative range. 

We have calculated the electrophilic and nucleophilic indexes of the ligands, and the results are shown in Table S6. Figure S6a presents a schematic representation of the changes in nucleophilic indexes of the ligands before and after deprotonation. The results showed that the nucleophilic index (*N*) value of the ligands increased dramatically after deprotonation.

This indicates that the deprotonation process increases the electron density of the ligands and significantly enhances their electron loss potential. These results suggest that the deprotonation process strongly influences the electronic properties of the ligands, including *E_HOMO_* values, energy gaps, electrostatic potentials, and nucleophilic indexes. These changes enable the deprotonated ligands to have stronger binding energies when binding with metal ions.

### 4.2. Differences in Binding Properties of Neutral Ligands

**Figure 8:**
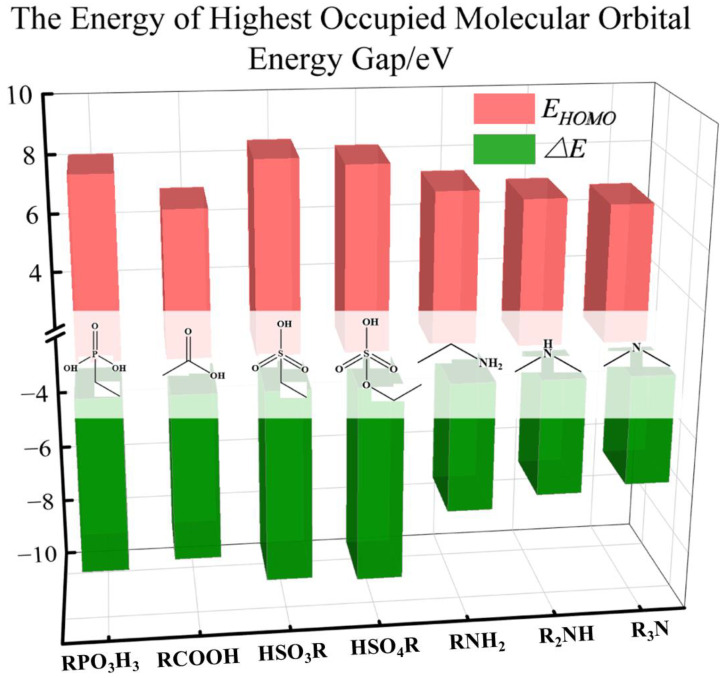
Bar chart of the highest occupied molecular orbital energy value (*E_HOMO_*) and energy gap of the neutral ligands.

The computational results from the previous chapter also indicated that the binding strengths of phosphorous acid and nitrogen ligands are higher than those of the other neutral ligands. We further investigated the mechanism behind this observation. Figure 8 presents the bar graph of the highest occupied molecular orbital (HOMO) energy values and energy gaps for the seven neutral ligands. The results show that the three nitrogen ligands, namely amine, diamine, and triamine, not only have the highest HOMO orbital values (at −6.20, −5.78, and −5.55 eV, respectively) but also have smaller energy gaps (at 7.22, 6.87, and 6.61 eV, respectively). Figure S6b shows the bar chart of the nucleophilicity of the neutral ligands. Compared with other ligands, the three nitrogen ligands exhibit stronger nucleophilic properties. These results suggest that they have higher electron loss capabilities and reactivity. Therefore, nitrogen ligands are typically excellent electron donors. The results are consistent with similar research findings, where nitrogen ligands with similar structures are often observed to be superior to oxygen ligands [12,40]. 

**Figure 9:**
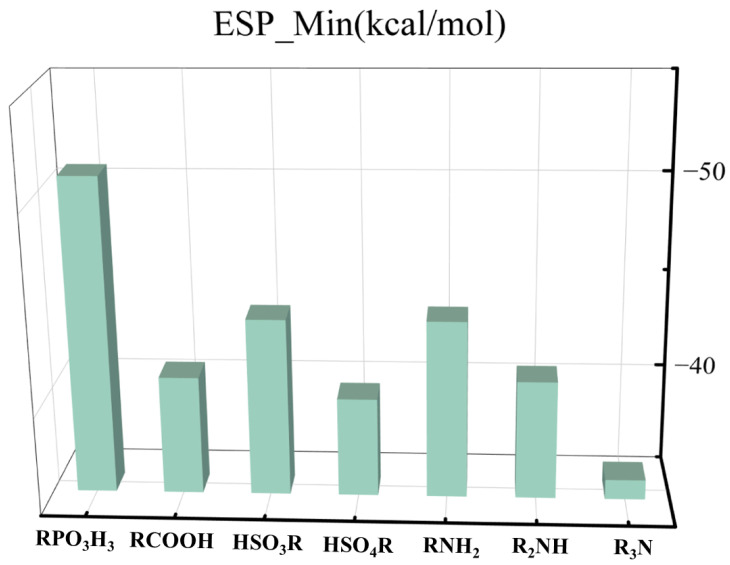
Bar chart of the minimum electrostatic potential values of the seven neutral ligands.

Figure 9 shows the histogram of the minimum electrostatic potential values for the seven neutral ligands. Among the non-deprotonated ligands, phosphoric acid has the most negative electrostatic potential (−50.3 kcal/mol), indicating the highest free electron density. Therefore, it also has a strong binding with metal ions.

To more accurately explore the binding mechanism between neutral ligands and metal ions, we conducted energy decomposition calculations on the interactions of the seven neutral ligands and one water molecule with copper and iron ions. The results are shown in Table S7. The results indicate that in all systems, electrostatic interactions make a decisive contribution to the binding strength. Therefore, there is no doubt that the main nature of the coordination bond between metal ions and organic ligands is electrostatic interaction. Dispersion effects also contribute to some systems, but they are second to electrostatic interaction. The main role of exchange repulsion is to offset the attractive effects produced by electrostatic and dispersion interactions.

**Figure 10:**
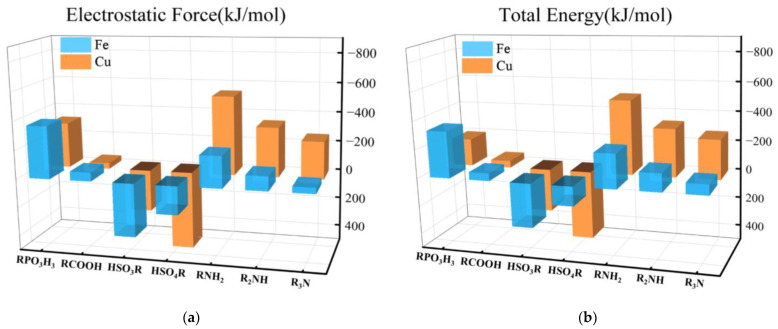
(**a**) Bar graph of the changes in electrostatic force when each of the seven neutral ligands replaces a water molecule from the Cu^2+^ and Fe^3+^ hydrate, (**b**) Bar graph of the changes in total energy when each of the seven neutral ligands replaces a water molecule from the Cu^2+^ and Fe^3+^ hydrates.

Figure 10a,b are bar graphs representing the increase in electrostatic forces and total energies between the seven neutral ligands with copper and iron ions compared to the value of one water molecule, respectively. The data reveals that the nitrogen ligands and the phosphite ligand exhibit pronounced electrostatic forces with the metal ions, significantly surpassing the other neutral ligands. Moreover, their total energies are also markedly higher than the remaining neutral ligands. A notable distinction is that the iron ion and the phosphite ligand demonstrate a stronger electrostatic force and total energy, whereas the copper ion and the nitrogen ligands present more potent electrostatic forces and total energies. This observation aligns with the selectivity trend discussed in the preceding section. It is important to note that in all seven instances, the exchange repulsion between copper ions and ligands exceeds that of iron ions. This discrepancy is likely attributable to the valence state of the metal ions.

### 4.3. Differences in Electrostatic Properties of Divalent and Trivalent Metal Ion Complexes

**Figure 11:**
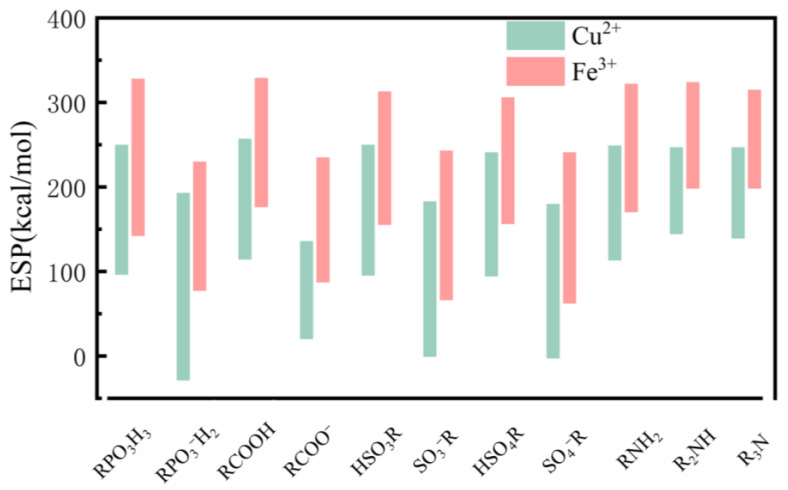
Comparison graph of the electrostatic potential of binding complexes with copper ions and iron ions.

We have also calculated the electrostatic potentials of the complexes formed by the metal ions and ligands, which are shown in Table S8. Figure 11 shows the electrostatic potential trend graph of Fe^3+^ and Cu^2+^ binding products in this study system. The results show that compared to divalent copper ion binding products, trivalent iron ion binding products have a more “positive” electrostatic potential. This can be explained by the fact that trivalent iron ions have a higher positive charge and can, therefore, bind electrons more strongly. This may be consistent with the result in energy decomposition calculations where divalent copper ions always have stronger exchange repulsion than trivalent iron ions.

**Figure 12:**
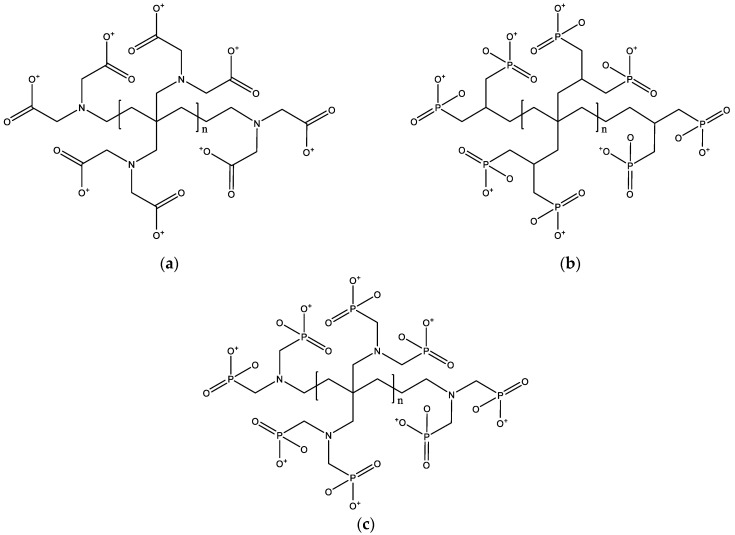
Predicted chelator structures: (**a**) Chelator structure suitable for divalent metal ions, (**b**) Chelator structure suitable for trivalent metal ions, (**c**) Chelator structure with strong binding capacity for all metal ions.

In summary, based on our series of computational results and mechanistic investigations, we selected the optimal ligands for each of the four metal ions, as shown in Table 1. According to the binding strength and structural characteristics of the ligands, we can divide them into edge ligands and bridging ligands. Finally, we speculated the chelating agent structures suitable for each metal ion based on the theoretical calculation results of this paper, which are shown in Figure 12. The chelating agent that can achieve strong binding with divalent metal ions may be an amino carboxylic acid chelating agent (such as Figure 12a), which is an EDTA-type chelating agent. So far, the EDTA-type chelating agent is still a very widely used type of chelating agent, which also verifies the rationality of our calculation results. Combined with the calculations in this paper, we have extended the structure of this class of chelators to give the strongest metal ion binding efficiency. For trivalent metal ions, the strong chelating agent type may be a chelating agent with more phosphorous acid ligands (such as Figure 12b). In addition, if it is necessary to use one chelator to achieve strong chelation of all types of metal ions, we recommend the amino phosphite chelator as in Figure 12c. It should be noted that with the change of chelating agent application scenarios, strong chelating agents may change. We hope that the results of this paper can help researchers and practitioners to choose appropriate chelating agents in microelectronic processes.

## 5. Conclusions

This paper presents the quantum chemical calculations of the binding process between eleven ligands and four metal ion hydrates (Cu^2+^, Ni^2+^, Al^3+^, Fe^3+^) and analyzes the molecular orbitals and electrostatic potential of the ligands to explore the main causes of the difference in ligand binding strength. According to our results, we found that firstly, the double-bonded oxygen of neutral oxygen ligands is more likely to be the binding site. Secondly, the binding of deprotonated ligands with four metal ions increased sharply, significantly better than all the non-deprotonated ligands, which may be due to the increase of HOMO orbital value, decrease of energy gaps, increase of nucleophilic indexes, and decrease of electrostatic potentials of deprotonated ligands. In addition, among the non-deprotonated ligands, nitrogen ligands and phosphorous acid ligands usually have stronger binding with metal ions, which may be because nitrogen ligands have higher HOMO orbital energy values, lower energy gaps and higher nucleophilicity indexes, and phosphorous acid ligands have the most negative electrostatic potential. Finally, the results of the energy decomposition calculations indicate that the primary nature of the coordination bond between metal ions and organic ligands is undoubtedly electrostatic interaction. The main role of exchange repulsion is to offset the attractive forces generated by electrostatic and dispersion interactions. Compared to trivalent iron ions, divalent copper ions usually have stronger covalent repulsion with ligands. However, the electrostatic interaction between copper ions and nitrogen ligands is exceptionally high, leading to a higher total binding energy. This may reflect its selectivity to some extent. These conclusions can guide us to find new and stronger chelating agents to solve the problem of metal ion contamination in microelectronic processes.

## Figures and Tables

**Table 1:** Summary of good ligands for each metal ion.

Metal Ions	Edge Position Ligand			Center Position Ligand		
Ni^2+^	carboxylic acid ion	phosphorous acid ion	sulfonic acid ion	amine	dimethylamine	trimethylamine
Cu^2+^	carboxylic acid ion	phosphorous acid ion	sulfonic acid ion	amine	dimethylamine	trimethylamine
Al^3+^	phosphorous acid ion	carboxylic acid ion	sulfonic acid ion	phosphorous acid	amine	dimethylamine
Fe^3+^	phosphorous acid ion	carboxylic acid ion	sulfonic acid ion	phosphorous acid	amine	dimethylamine

## Data Availability

Data are contained within the article.

## Supplementary Materials

The following supporting information can be downloaded at: https://www.mdpi.com/article/10.3390/molecules29020308/s1. Figure S1: Geometrically optimized structure of four metal ionic hydrates; Table S1: Table of theoretical and calculated values for metal ion coordination bond lengths; Figure S2: Computationally simulated geometries of binding products generated by the binding of three metal ions to eleven ligands with their different sites; Table S2: Table of metal ions and ligands binding enthalpies (eV); Table S3: Table of metal ions and ligands binding energies (eV); Figure S3: (a) Bar chart of the binding enthalpies and binding energies between nickel ions and four ligands at single and double bond oxygen sites, (b) Bar chart of the binding enthalpies and binding energies between aluminum ions and four ligands at single and double bond oxygen sites, (c) Bar chart of the binding enthalpies and binding energies between ferrous ions and four ligands at single and double bond oxygen sites; Table S4: Table of ligands frontier molecular orbital values and energy gap (eV); Table S5: Ligands electrostatic potential range table (kcal/mol); Figure S4: Electrostatic potential diagrams of the three nitrogen ligands (The blue point in the graph indicates the point of electrostatic potential minimum); Figure S5: Local electrophilic indexes and nucleophilic indexes(e*eV) plots for eleven ligands, where the atoms possessing the strongest nucleophilic indices for each ligand have been bolded; Table S6: The electrophilicity ω index and nucleophilicity *N* index(eV); Figure S6: (a) Graph of the change in nucleophilic indexes of ligands before and after deprotonation, (b) Histogram of the nucleophilic indexes of the neutral ligand; Table S7: Energy decomposition data based on molecular force field for the coordination bonds of copper ions and iron ions with neutral ligands (KJ/mol); Table S8: The electrostatic potential range of the complexes (kcal/mol).
