# Supplementary material for: DFT Exploration of Metal Ion–Ligand Binding: Toward Rational Design of Chelating Agent in Semiconductor Manufacturing
Source: Molecules. 2024 Jan 8;29(2):308. doi: 10.3390/molecules29020308 (PMC10819218; doi:10.3390/molecules29020308)
Supplement: Supplementary file 1 [file molecules-29-00308-s001.zip › molecules-2787171-supplementary.pdf]

1. Structural optimization

The optimized structures of metal ion hydrates are shown in Figure S1. After structural optimization using the computational method described in section 2 of the main text, the four types of metal ion hydrates present regular polygonal structures. Table S1 lists the calculated and the theoretical bond lengths of these hydrates.

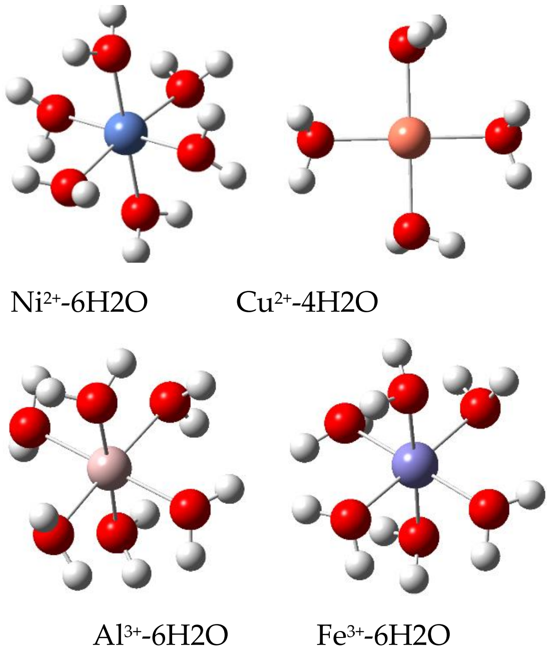

Figure S1. Geometrically optimized structure of four metal ionic hydrates.

Table S1. Table of theoretical and calculated values for metal ion coordination bond lengths.

Metal ions	Item		
	**Coordination number**	**Theoretical bond length (Å)**	**Calculate bond length (Å)**
Ni^{2+}	6	2.06	2.07
Cu^{2+}	4	1.95	1.97
Al^{3+}	6	1.92	1.91
Fe^{3+}	6	2.05	2.02

The optimized structures of the substitution complexes formed by the metal ions and ligands are shown in Figure S2. The relative positions of the ligand's coordinating atoms and the metal atoms remain essentially unchanged.

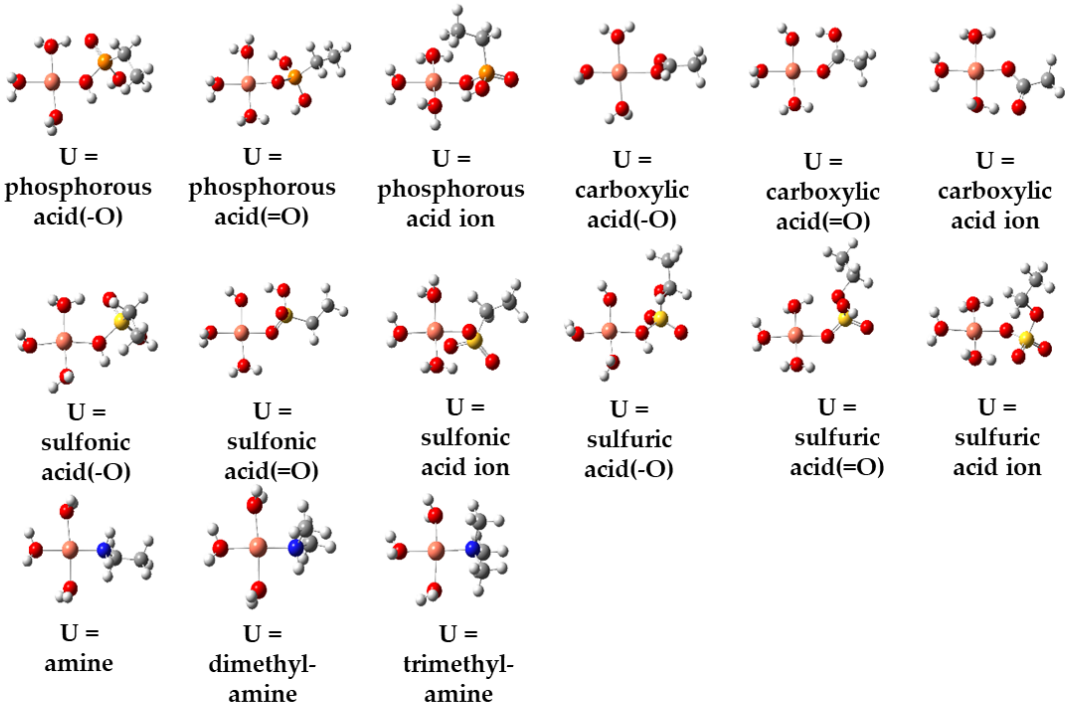

Cu^{2+}-3H2O-U

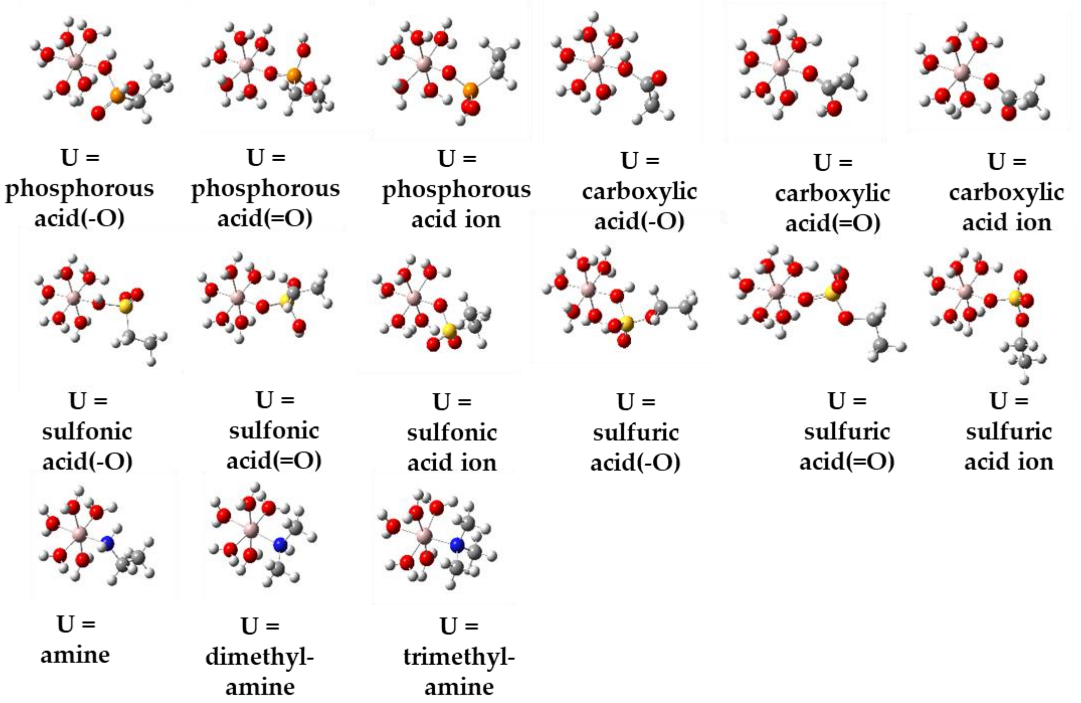

Al^{3+}-5H2O-U

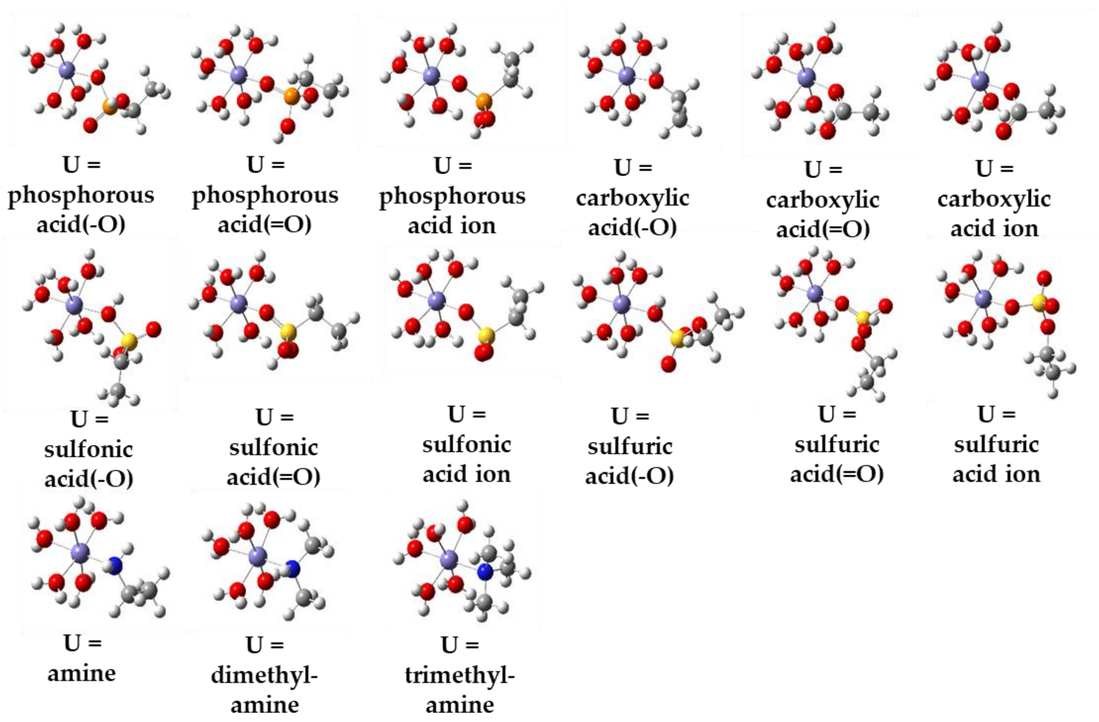

Fe^{3+}-5H2O-U

Figure S2. Computationally simulated geometries of binding products generated by the binding of three metal ions to eleven ligands with their different sites.

2. Binding strength analysis

Tables S2 and S3 summarize the binding energies and binding enthalpies of the substitution processes involving four types of metal ion hydrates and ligands.

Table S2. Table of metal ions and ligands binding enthalpies (eV).

Metal ions	Ligand					
	phosphorous acid(=O)	phosphorous acid(-O)	phosphorous acid ion	carboxylic acid(=O)	carboxylic acid(-O)	carboxylic acid ion
Ni^{2+}	-0.234	0.070	-1.192	-0.067	0.165	-1.302
Cu^{2+}	-0.441	-0.096	-1.219	-0.127	-0.097	-1.860
Al^{3+}	-0.593	0.010	-2.124	-0.0020	0.556	-2.020
Fe^{3+}	-0.669	-0.052	-2.303	-0.092	0.503	-2.220
**Metal ions**	**Ligand**					
	sulfonic acid(=O)	sulfonic acid(-O)	sulfonic acid ion	sulfuric acid(=O)	sulfuric acid(-O)	sulfuric acid ion
Ni^{2+}	0.030	0.143	-0.928	0.193	0.249	-0.611
Cu^{2+}	0.054	0.219	-0.942	0.151	0.452	-0.899
Al^{3+}	-0.033	0.406	-1.415	0.220	0.616	-1.051
Fe^{3+}	-0.106	0.408	-1.530	0.183	0.595	-1.217
**Metal ions**	**Ligand**					
	amine	dimethylamine	trimethylamine			
Ni^{2+}	-0.530	-0.496	-0.364			
Cu^{2+}	-0.890	-0.885	-0.794			
Al^{3+}	-0.452	-0.399	-0.221			
Fe^{3+}	-0.591	-0.555	-0.412			

Table S3. Table of metal ions and ligands binding energies (eV).

Metal ions	Ligand					
	phosphorous acid(=O)	phosphorous acid(-O)	phosphorous acid ion	carboxylic acid(=O)	carboxylic acid(-O)	carboxylic acid ion
Ni^{2+}	-0.222	0.072	-1.177	-0.079	0.177	-1.329
Cu^{2+}	-0.516	-0.112	-1.200	-0.113	-0.104	-1.804
Al^{3+}	-0.683	0.024	-2.086	0.007	0.659	-1.974
Fe^{3+}	-0.804	-0.096	-2.320	-0.140	0.496	-2.248

Metal ions	Ligand					
	sulfonic acid(=O)	sulfonic acid(-O)	sulfonic acid ion	sulfuric acid(=O)	sulfuric acid(-O)	sulfuric acid ion
Ni^{2+}	0.030	0.234	-0.928	0.193	0.299	-0.611
Cu^{2+}	0.114	0.325	-0.851	0.158	0.517	-0.908
Al^{3+}	-0.012	0.546	-1.393	0.152	0.704	-1.109
Fe^{3+}	-0.092	0.520	-1.515	0.113	0.679	-1.291

Metal ions	Ligand		
	amine	dimethylamine	trimethylamine
Ni^{2+}	-0.530	-0.496	-0.364
Cu^{2+}	-0.881	-0.846	-0.746
Al^{3+}	-0.399	-0.318	-0.087
Fe^{3+}	-0.604	-0.591	-0.450

Figure S3 presents the bar graphs of the binding enthalpies and binding energies of $Ni^{2+,}$ Al^{3+}, Fe^{3+} with four ligands at their single-bond oxygen and double-bond oxygen sites. Stronger binding between ligand’s double-bond oxygen sites and metal ions has been observed.

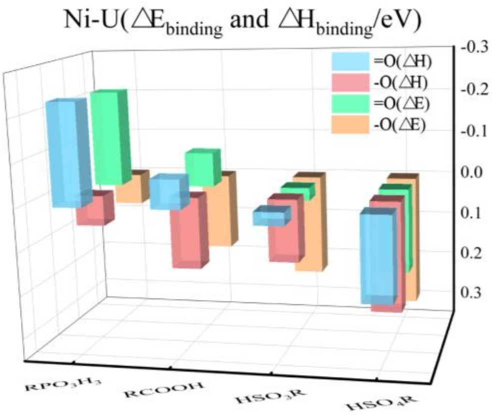

(**a**)

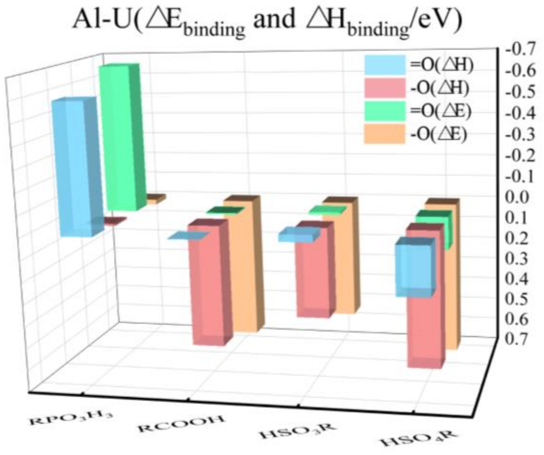

(**b**)

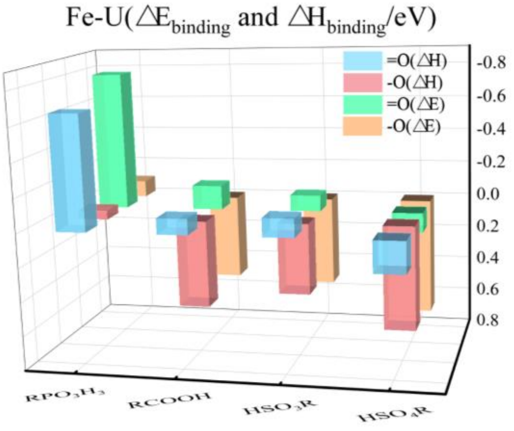

(c)

Figure S3. (a) Bar chart of the binding enthalpies and binding energies between nickel ions and four ligands at single and double bond oxygen sites, (b) Bar chart of the binding enthalpies and binding energies between aluminum ions and four ligands at single and double bond oxygen sites, (c) Bar chart of the binding enthalpies and binding energies between ferrous ions and four ligands at single and double bond oxygen sites.

3. Binding Mechanism Exploration

3.1 Frontline molecular orbital analysis

Table S4 displays the frontier molecular orbital values and energy gap values of the eleven ligands studied in this research.

Table S4. Table of ligands frontier molecular orbital values and energy gap (eV).

Ligand	phosphorous acid	phosphorous acid ion	carboxylic acid	carboxylic acid ion	sulfonic acid	sulfonic acid ion
E_{LOMO}	0.40	1.06	-0.52	1.49	0.13	1.43
E_{HOMO}	-7.66	-6.06	-7.39	-5.23	-8.28	-5.99
$\triangle E$	8.06	7.14	6.86	6.72	8.41	7.41
**Ligand**	**sulfuric acid**	**sulfuric acid ion**	**amine**	**dimethylamine**	**trimethylamine**	
E_{LOMO}	-0.26	1.35	1.02	1.10	1.06	
E_{HOMO}	-8.43	-6.38	-6.20	-5.78	-5.55	
$\triangle E$	8.16	7.73	7.22	6.87	6.61	

3.2 Electrostatic potential analysis of the ligands

Table S5 presents the electrostatic potentials of the eleven ligands studied in this paper. The results show that the electron losing potential of the four acid ligands increases after deprotonation.

Table S5. Ligands electrostatic potential range table (kcal/mol).

**Ligand**	**phosphorous acid**	**phosphorous acid ion**	**carboxylic acid**	**carboxylic acid ion**	**sulfonic acid**	**sulfonic acid ion**
ESP	-51~58	-155~-35	-41~53	-165~-65	-44~70	-145~-39
**Ligand**	**sulfuric acid**	**sulfuric acid ion**	**amine**	**dimethylamine**	**trimethylamine**	
ESP	-40~74	-138~-33	-44~27	-41~27	-36~10	

Figure S4 displays the electrostatic potential distribution diagrams of three nitrogen ligands. The minimum points of the electrostatic potential are distributed on the van der Waals surface of the nitrogen atom.

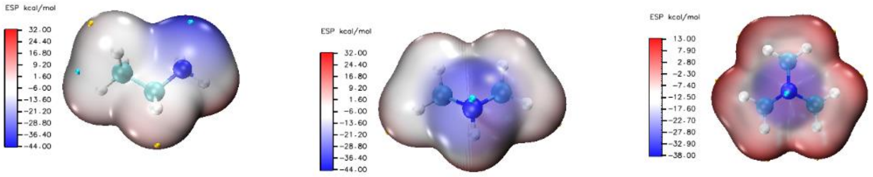

amine dimethylamine trimethylamine

Figure S4. Electrostatic potential diagrams of the three nitrogen ligands (The blue point in the graph indicates the point of electrostatic potential minimum).

3.3 The electrophilicity ω index and the nucleophilicity N index.

Figure S6 shows the electrophilicity and nucleophilicity indexes of each atom in the ligands. Based on these results, the most likely atoms to undergo electrophilic/nucleophilic reactions on the ligands can be easily identified. Table S6 presents the global electrophilicity and nucleophilicity indexes of the molecules. These results can be used to compare the electrophilic/nucleophilic potentials of different molecules.

**phosphorous acid**		
Atom	Electrophilicity	Nucleophilicity
1(C)	0.01714	0.02975
2(H)	0.02538	0.05188
3(H)	0.01489	0.02094
4(H)	0.01489	0.02094
5(C)	0.02122	0.0315
6(H)	0.02528	0.03589
7(H)	0.02528	0.03589
8(P)	0.0531	0.19048
**9(O)**	**0.03376**	**0.5765**
10(O)	0.04592	0.20474
11(H)	0.09402	0.06679
12(O)	0.04592	0.20474
13(H)	0.09402	0.06679

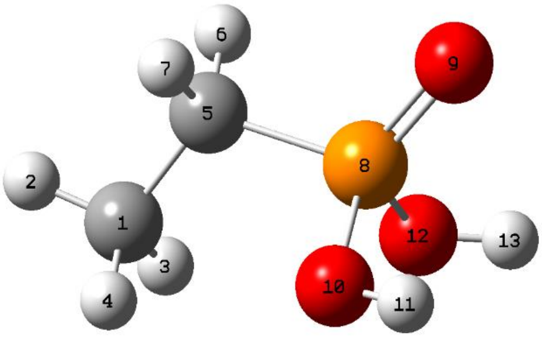

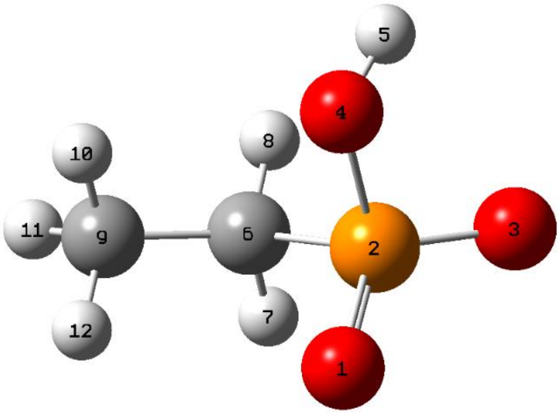

**phosphorous acid ion**		
Atom	Electrophilicity	Nucleophilicity
**1(O)**	**0.01043**	**1.93949**
2(P)	0.01204	1.14704
3(O)	0.0089	1.81089
4(O)	0.01493	0.56755
5(H)	0.03419	0.33619
6(C)	0.00934	0.59082
7(H)	0.01366	0.31035
8(H)	0.01348	0.39163
9(C)	0.01232	0.24434
10(H)	0.01172	0.18771
11(H)	0.01837	0.39544
12(H)	0.00988	0.17441

carboxylic acid		
Atom	Electrophilicity	Nucleophilicity
1(C)	0.0393	0.12694
2(H)	0.02552	0.07346
3(H)	0.04826	0.10689
4(H)	0.04818	0.10693
5(C)	0.12444	0.20179
**6(O)**	**0.1223**	**0.69965**
7(O)	0.06169	0.26838
8(H)	0.0332	0.09992

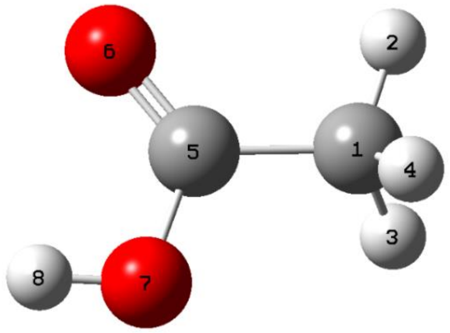

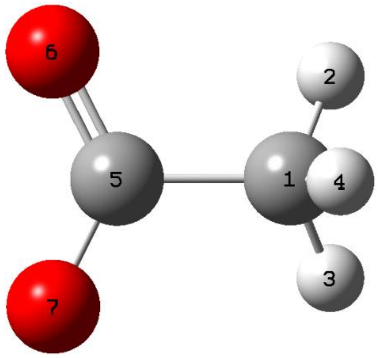

carboxylic acid ion		
Atom	Electrophilicity	Nucleophilicity
1(C)	0.08291	0.45557
2(H)	0.05306	0.5058
3(H)	0.05248	0.50248
4(H)	0.16671	0.4635
5(C)	0.05894	1.14996
6(O)	0.06459	3.2621
**7(O)**	**0.06434**	**3.27521**

sulfonic acid		
Atom	Electrophilicity	Nucleophilicity
1(S)	0.05888	0.09766
**2(O)**	**0.06067**	**0.24065**
3(O)	0.04875	0.19744
4(O)	0.0806	0.07822
5(C)	0.03201	0.03804
6(H)	0.0349	0.03876
7(H)	0.02359	0.02798
8(C)	0.02197	0.01972
9(H)	0.01829	0.01782
10(H)	0.02146	0.03553
11(H)	0.01854	0.01492
12(H)	0.15716	0.03769

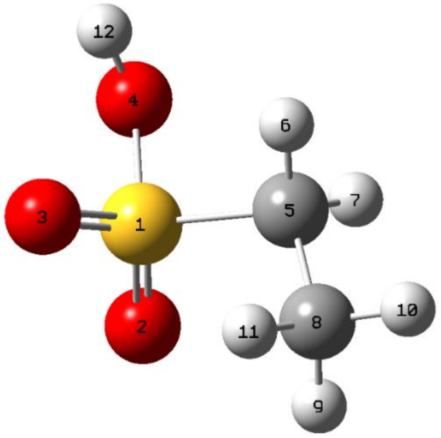

sulfonic acid ion		
Atom	Electrophilicity	Nucleophilicity
1(S)	0.01099	0.87152
**2(O)**	**0.01218**	**1.92557**
3(O)	0.01218	1.92557
4(O)	0.01288	1.93539
5(C)	0.01678	0.25209
6(H)	0.02518	0.24299
7(H)	0.02518	0.24299
8(C)	0.02771	0.14468
9(H)	0.02214	0.11684
10(H)	0.04477	0.30915
11(H)	0.02214	0.11684

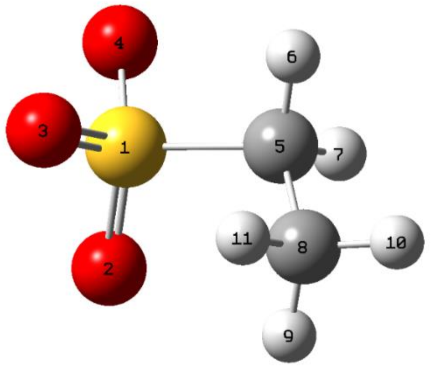

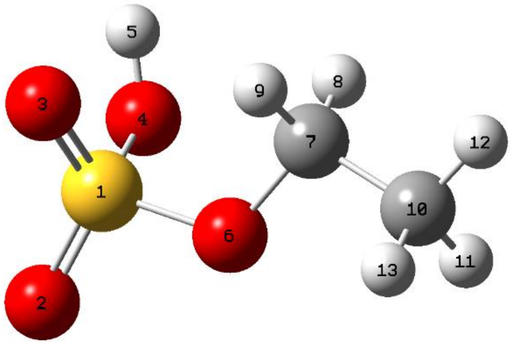

sulfuric acid		
Atom	Electrophilicity	Nucleophilicity
1(S)	0.08109	0.05046
**2(O)**	**0.08059**	**0.12344**
3(O)	0.06278	0.0956
4(O)	0.08725	0.04875
5(H)	0.15395	0.02198
6(O)	0.0472	0.07944
7(C)	0.03301	0.02177
8(H)	0.02971	0.02606
9(H)	0.02186	0.02569
10(C)	0.02054	0.01405
11(H)	0.0156	0.01467
12(H)	0.03535	0.01863
13(H)	0.01721	0.01452

sulfuric acid ion		
Atom	Electrophilicity	Nucleophilicity
1(S)	0.00653	0.85314
**2(O)**	**0.00679**	**1.854**
3(O)	0.00679	1.854
4(O)	0.00829	1.80949
5(C)	0.00947	0.10751
6(H)	0.01064	0.11296
7(H)	0.01064	0.11296
8(O)	0.00372	0.3503
9(C)	0.02006	0.12463
10(H)	0.02953	0.20196
11(H)	0.01821	0.11931
12(H)	0.01821	0.11931

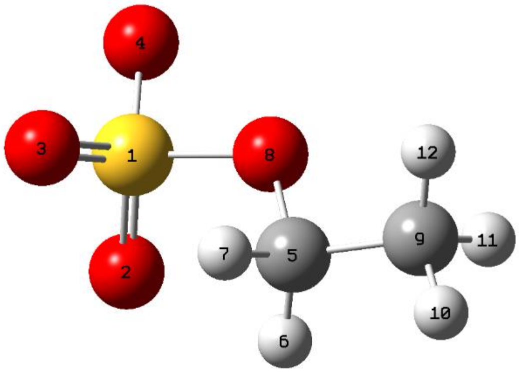

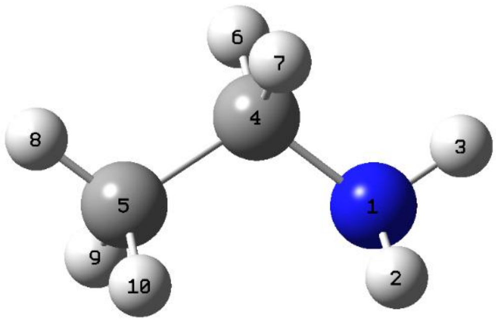

amine		
Atom	Electrophilicity	Nucleophilicity
**1(N)**	**0.02501**	**1.10716**
2(H)	0.03678	0.3101
3(H)	0.04148	0.31243
4(C)	0.01745	0.21851
5(C)	0.01668	0.09876
6(H)	0.01733	0.17745
7(H)	0.021	0.34238
8(H)	0.0225	0.13771
9(H)	0.01269	0.10785
10(H)	0.01669	0.08891

dimethylamine		
Atom	Electrophilicity	Nucleophilicity
**1(N)**	**0.0134**	**1.09637**
2(H)	0.0314	0.31415
3(C)	0.01765	0.23165
4(H)	0.01903	0.17869
5(H)	0.01446	0.19018
6(H)	0.01638	0.31848
7(C)	0.01765	0.23165
8(H)	0.01446	0.19018
9(H)	0.01903	0.17869
10(H)	0.01638	0.31848

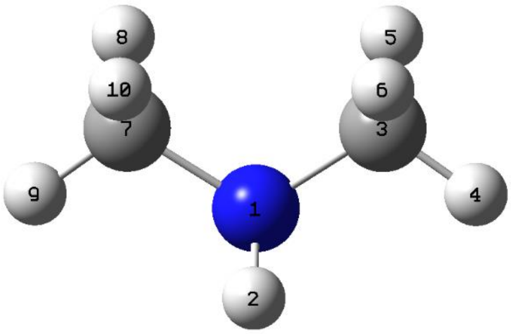

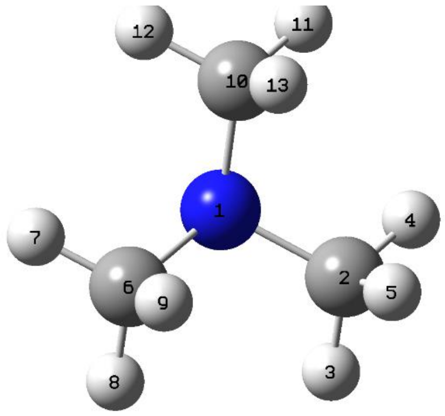

trimethylamine		
Atom	Electrophilicity	Nucleophilicity
**1(N)**	**0.00309**	**0.97905**
2(C)	0.01293	0.19634
3(H)	0.01475	0.16565
4(H)	0.01475	0.16565
5(H)	0.01249	0.29077
6(C)	0.01293	0.19631
7(H)	0.01475	0.16563
8(H)	0.01475	0.16563
9(H)	0.01249	0.29076
10(C)	0.01293	0.19631
11(H)	0.01475	0.16563
12(H)	0.01475	0.16563
13(H)	0.01249	0.29076

Figure S5. Local electrophilic indexes and nucleophilic indexes(e*eV) plots for eleven ligands, where the atoms possessing the strongest nucleophilic indices for each ligand have been bolded.

Table S6. The electrophilicity ω index and nucleophilicity N index(eV).

**Ligand**	**phosphorous acid**	**phosphorous acid ion**	**carboxylic acid**	**carboxylic acid ion**	**sulfonic acid**	**sulfonic acid ion**
ω	0.511	0.169	0.503	0.543	0.577	0.232
N	1.537	8.096	1.684	9.615	0.844	8.084
**Ligand**	**sulfuric acid**	**sulfuric acid ion**	**amine**	**dimethylamine**	**trimethylamine**	
ω	0.686	0.149	0.228	0.180	0.168	
N	0.555	7.619	2.901	3.249	3.434	

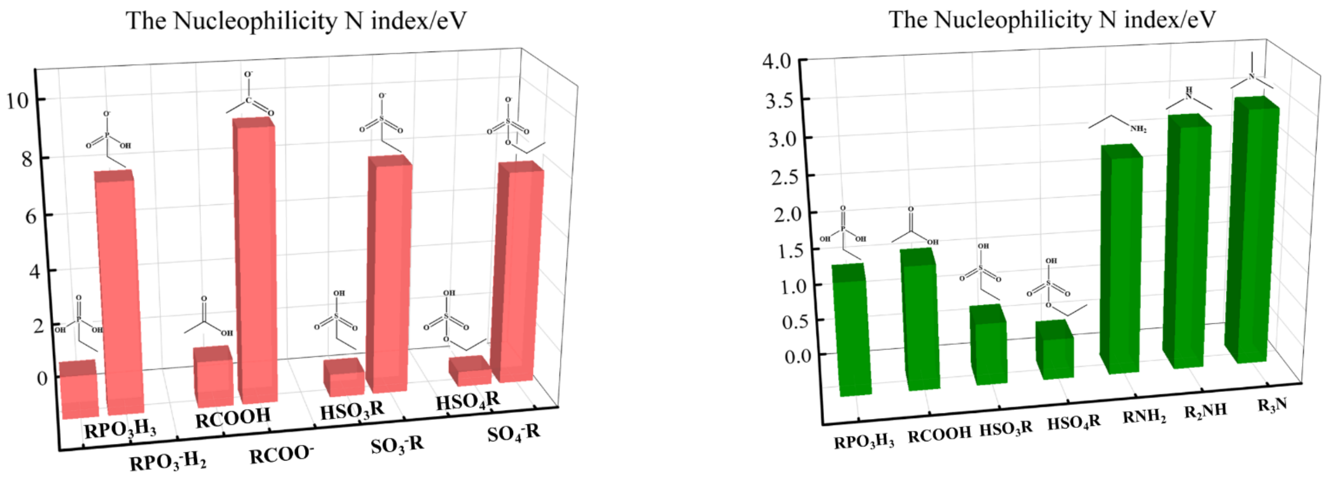

(**a**) (**b**)

Figure S6. (a) Graph of the change in nucleophilic indexes of ligands before and after deprotonation, (b) Histogram of the nucleophilic indexes of the neutral ligand.

3.4 Energy Decomposition Analysis of Cu^{2+} and Fe^{3+} Complexes Based on Molecular Force Field

Table S7 presents the energy decomposition results of the coordination bonds of Cu^{2+} and Fe^{3+} with the eleven ligands.

Table S7. Energy decomposition data based on molecular force field for the coordination bonds of copper ions and iron ions with neutral ligands (KJ/mol).

Ligand	phosphorous acid	carboxylic acid	sulfonic acid	sulfuric acid	amine
Electrostatic (Cu)	-508.77	-234.67	91.83	342.52	-732.69
Electrostatic (Fe)	-579.01	-275.84	155.22	-16.68	-429.77
Repulsive (Cu)	300.35	147.37	161.93	78.61	176.61
Repulsive (Fe)	138.6	112.06	16.63	12.85	80.87
Dispersion (Cu)	-48.17	-26.77	-24.91	-13.1	-20.81
Dispersion (Fe)	-38.11	-39.86	-13.25	-10.29	-37.74
Total (Cu)	-256.59	-114.08	228.85	408.03	-576.89
Total (Fe)	-478.52	-203.64	158.6	-14.13	-386.64

Ligand	dimethyl amine	trimethylamine	water
Electrostatic (Cu)	-532.95	-449.87	-195.71
Electrostatic (Fe)	-312.81	-254.91	-213.78
Repulsive (Cu)	157.6	131.86	146.4
Repulsive (Fe)	77.8	80.29	89.67
Dispersion (Cu)	-20.74	-19.07	-17.59
Dispersion (Fe)	-40.55	-48.96	-25.88
Total (Cu)	-396.09	-337.08	-66.9
Total (Fe)	-275.56	-223.58	-149.99

3.5 Analysis of the electrostatic potential of bound complexes.

Table S8 presents the electrostatic potentials of the products resulting from the substitution processes involving four types of metal ions and eleven ligands.

Table S8. The electrostatic potential range of the complexes (kcal/mol).

**Metal ions**	**Ligand**					
	**phosphorous acid**	**phosphorous acid ion**	**carboxylic acid**	**carboxylic acid ion**	**sulfonic acid**	**Sulfonic acid ion**
Ni^{2+}	96~237	-4~159	101~224	15~159	96~221	12~163
Cu^{2+}	96~250	-29~193	114~257	20~136	95~250	-1~183
Al^{3+}	138~326	69~244	169~322	75~251	150~313	81~257
Fe^{3+}	142~328	77~230	176~329	87~235	155~313	66~243
**Metal ions**	**Ligand**					
	**sulfuric acid**	**sulfuric acid ion**	**amine**	**dimethylamine**	**trimethylamine**	
Ni^{2+}	98~222	17~168	105~230	130~228	128~229	
Cu^{2+}	94~241	-3~180	113~249	144~247	139~247	
Al^{3+}	138~313	56~257	169~332	194~332	194~326	
Fe^{3+}	156~306	62~241	170~322	198~324	198~315	
